# Isn’t There an App for That? The Role of Smartphone and Tablet Applications for Asthma Education and Self-Management in Adolescents

**DOI:** 10.3390/children8090786

**Published:** 2021-09-09

**Authors:** Antonia O’Connor, Andrew Tai, Kristin Carson-Chahhoud

**Affiliations:** 1 Respiratory and Sleep Department, Women’s and Children’s Hospital, 72 King Williams Road, North Adelaide, SA 5006, Australia; andrew.tai@sa.gov.au; 2 Robinson Research Institute, University of Adelaide, Ground Floor, Norwich Centre, 55 King William Road, North Adelaide, SA 5006, Australia; 3 Adelaide Medical School, University of Adelaide, Adelaide Health and Medical Sciences Building, Corner of North Terrace & George Street, Adelaide, SA 5000, Australia; Kristin.Carson-chahhoud@unisa.edu.au; 4 Translational Medicine and Technology Group, Australian Centre for Precision Health, University of South Australia, Level 8 South SAHMRI Building, North Terrace, Adelaide, SA 5000, Australia; 5 Australian Centre for Precision Health, South Australian Health and Medical Research Institute, North Terrace, Adelaide, SA 5000, Australia

**Keywords:** bronchial asthma, asthma impact, adolescents, self-management, mobile health, technology, smartphone, tablet

## Abstract

Asthma is one of the most common chronic diseases worldwide, with a substantial proportion of the asthma population being children and adolescents. Self-management is recognized as a key component to asthma management, with multiple international guidelines emphasizing the need for adequate self-management skills for good asthma control. Unfortunately, the uptake amongst young people and adolescents is low, with often suboptimal engagement to self-management education and skills contributing to poor adherence to medication as well as poor perception of asthma symptoms. Innovative solutions to deliver education and self-management to adolescents are clearly needed. mHealth is the use of mobile devices such as smartphones and tablet devices to improve healthcare and has been used in multiple chronic diseases. This review articles explores the current use of mHealth in asthma, specifically smartphone and tablet applications as a generation-appropriate, accessible delivery modality for provision of asthma education and self-management interventions in adolescents. Current evidence gaps are also highlighted, which should be addressed in future research.

## 1. Introduction

Asthma is one of the most prevalent chronic diseases worldwide, with an estimated 330 million people diagnosed [1]. Despite the increasing knowledge surrounding pathophysiology and treatment of the disease, prevalence is still estimated to be increasing, with 100 million more people anticipated to have asthma by 2025 [2]. Children and adolescents make up a substantial proportion of this group, with the paediatric population having a higher incidence and prevalence than adults [3]. Children and adolescents who have poorly controlled asthma contribute significantly to health care systems and government costs with an estimated 5.35 billion dollars per year in the United States alone spent on managing the disease within this population [4]. Asthma self-management programs have been shown to assist in achieving asthma control, improving quality of life, reducing health care utilisation and are recommended in management guidelines [5,6,7,8,9]. However, among young people and adolescents with asthma, there is often poor adherence to medication and a poor perception of symptoms, which may be a reflection of suboptimal engagement in asthma self-management and education [10,11,12]. Innovative solutions for delivery of education and self-management in this age group have been more recently explored via the use of smartphone and tablet devices. The use of these technologies to enhance health and chronic disease management is known as mobile health (mHealth) [13]. This review article aims to be a rapid review of the current evidence and use of smartphone and tablet devices as a specific modality of mHealth, as a generation-appropriate and readily available delivery modality for self-management among adolescents, to identify current evidence gaps which should be addressed in future research.

## 2. Asthma Self-Management

Whilst there are many pharmacological agents proven to be efficacious in asthma, self-management programs and self-management skills are also recognised as a key component in disease management. Self-management can be defined as ‘the tasks that individuals must undertake to live with one or more chronic conditions’ and is an important component in all chronic disease management [14]. Broadly, self-management programs aim to overall change behaviours, and develop skills specific to the condition with techniques including improving knowledge of the disease, encouraging active participation in care, empowering the patient and supporting adherence to strategies which may prevent complications and symptoms [15,16,17]. Asthma self-management programs commonly include information regarding asthma pathophysiology, training on self-monitoring of symptoms, information regarding avoidance of triggers, as well as frequent medication and device technique reviews [6,8,9,15,18]. As well as education, guided self-management should also include a written action plan which is personalised and be regularly reviewed by health professionals [9,19]. The personalised plan supports self-management for those with asthma, containing information such as symptom recognition, instructions on use of prescribed inhalers, and immediate management guidance for an acute exacerbation such as inhaler use and triggers to seek urgent health care review [19]. Multiple randomised controlled trials have shown that self-management education programs are effective in the paediatric asthma population in improving lung function, decreasing missed school days, as well as decreasing unplanned healthcare visits [20,21,22,23]. The evidence for self-management programs is also robust in adults with asthma, with a Cochrane systematic review reporting reduced hospitalisations, reduced urgent healthcare utilisation, reduced missed work or school days and improved quality of life in those with self-management education involving interventions such as asthma plans, self-monitoring of symptoms and frequent review by health professionals [24]. To be effective, however, sufficient time is required from trained healthcare professionals to help deliver self-management education, and patients must be receptive to the development of these skills, which may be difficult among the adolescent cohort.

## 3. Adolescents and Self-Management

Adolescence, in which there are significant physical, emotional, and social changes in the transition to adulthood, makes for a particularly challenging period for the self-management of chronic diseases [12,25,26]. It is a time of increasing independence and desire for autonomy. However, this is juxtaposed with an ongoing dependence on health professionals and medications. As such, adolescents may experience emotions such as resentment towards their asthma and their ‘sick’ role [12,26]. Adherence to treatment may be affected by competing interests in their day-to-day lives including schooling, new opportunities for employment and social activities. Adolescents are estimated to only take up to 50% of their prescribed medications [26,27]. This transition period also marks a time where there is an increase in risk-taking behaviours, among which smoking cigarettes is common [28]. Adolescents may also be embarrassed or feel different to their peers if needing to take reliever medications in public and hence be more likely to have untreated symptoms [12]. In a cross-sectional study of 126 adolescents with asthma, these indeed were some of the most common barriers to asthma self-management in which it was reported that there was an unwillingness to give up behaviours which doctors had asked them to, forgetfulness of medications, and a sense of ‘trying to forget’ their diagnosis were commonly identified [27]. Although effective communication and education remains crucial in this period, traditional self-management programs may not be as successful [5]. With the known decline of adherence to effective disease management during adolescence, it is clear that there is a need for innovative strategies to approach asthma self-management education in the adolescent population [29]. Positively, childhood and early adolescence has been shown to be an optimal time for skills acquisition and reinforcement learning [30,31]. Combined with the increasing drive for independence, there is an opportunity for engagement and ownership over asthma self-management during this period.

## 4. Smartphone and Tablet Application Use in Self-Management and Education of Chronic Diseases

Information and communication technologies (ICTs) have been increasingly used for medical purposes as a strategy to improve communication between patients and health care professionals, as well as to provide information and education to patients, and track health data across healthcare professions [32,33]. ICTs are defined as tools that are able to ‘communicate, manipulate and store data by electronic means’ and include both smartphone and tablet devices [34]. The use of these mobile devices to improve healthcare and public health is known as ‘mHealth’ [33]. These devices can remind patients of appointments with text messages, but most commonly, they are used in mHealth through mobile applications (apps) [5,15]. Telemedicine has also been employed, allowing improved patient access to support and an encouragement of adherence [26,35].

Over the past decade, mHealth has been adopted by multiple chronic diseases for behaviour change and self-management. These chronic diseases include diabetes mellitus, asthma and cancer and employ strategies such as encouraging increasing frequency of blood glucose monitoring, which has been shown to be effective in behaviour change for both adults and adolescents [36,37]. In addition, there are numerous studies identifying improved outcomes such as decreased healthcare utilisation when self-management is delivered via telemedicine [38,39]. Smartphones and tablets offer a convenient, easily accessible, cost-effective mode of self-management tool delivery with the benefit of being capable of patient-specific tailoring, and is readily updated as the evidence changes [5,26,40,41]. As technology continues to rapidly evolve and improve, mHealth and the use of smartphone and tablet technology will no doubt be a communication disrupter to the delivery of self-management tools for education.

Health-related mobile apps are rapidly increasing, with a recent digital health trend report stating there are over 325,000 apps available worldwide currently, and more than 90,000 new health apps were added to major smartphone app stores in 2020 alone [42,43,44]. The focus on health-related mobile apps has also shifted, with an increasing focus on the management of specific health conditions rather than overall wellness [44]. The increasing use of ICTs for management of chronic illnesses and for healthcare promotion is likely multifactorial, with an increasing number and quality of devices and apps, a decrease in the cost of devices and mobile data, as well as a population which is becoming more technology-proficient all contributing [15,45].

## 5. Smartphone and Tablet Applications in Asthma Self-Management and Education

As described previously, asthma self-management education is known to be an important component of management. However exactly how delivery should occur is not explicit in the guidelines. Whilst information delivered face-to-face and on paper-based resources will always have its place, the convenience and accessibility has allowed self-management interventions and education delivered via smartphone and tablet devices to become increasingly attractive and acceptable options [40]. Apps created for asthma include varied combinations of disease information provision, social support and self-management tools such as asthma action plans, symptom diaries, medication reminders and information regarding environmental triggers (e.g., pollen count) [5]. It may also allow for self-entry of data to allow the clinicians involved to monitor their health remotely to ensure timely intervention if required [46].

Recently, there has been multiple systematic reviews surrounding the use of mHealth and technology-based interventions that have shown improved adherence and quality of life; however, there have been few which have reviewed the use of smartphone and tablet apps specifically comparative to usual care [47,48,49,50,51,52,53]. In 2013, Belisario et al. identified two randomised controlled trials which compared mobile-based self-management to paper-based self-management but were unable to draw any conclusions in their systematic review, with insufficient studies and heterogeneity within the included studies. Since then, there have been two systematic reviews identified examining the use of smartphone and tablet applications in asthma self-management and its beneficial impact on outcomes [18]. A meta-analysis conducted in 2016 by Hui et al. reviewed mobile apps as a delivery method for self-management support for adult asthmatics, showing improved asthma control (using the Asthma Control Questionnaire), as well as improved quality of life over 6–30 months in half of the interventions [54]. In 2017, Farzandipour et al. identified 10 studies reviewing the effect of mobile apps on asthma self-management with most of the included studies (90%) reporting a positive impact of the intervention on outcomes assessed. Overall, there were statistically significant improvements in asthma control as well as a positive impact on quality of life, though mixed effects on medication adherence and healthcare utilisation were observed [55].

## 6. Can Smartphone and Tablet Applications Improve Education and Self-Management in the Adolescent Asthma Population and Where Are the Current Gaps in the Evidence?

The use of smartphone and tablet applications for asthma is particularly promising as a self-management and educational tool in the adolescent age group. Adolescents not only have increasing access to smartphone and tablet devices, but they also use the internet more frequently than ever before. In a survey of teenagers in the United States in 2018, 95% of youths aged 13–17 years reported that they either had a smartphone or could access one, and 45% of teenagers reported almost constant use of the internet [56]. This internet usage had almost doubled since the 2015 survey [56]. Therefore, with the ability of smartphones, tablets and the internet to engage this demographic, it is logical that health interventions should be tailored towards these modalities to provide a generation-appropriate and accessible platform [57], especially for disease self-management.

For asthma self-management, engagement is particularly important at this stage, as strong beliefs regarding their asthma and medication usage will be formed during adolescence. These beliefs may be long-lasting, and as previously mentioned, adherence rates also decrease in this time [58]. Therefore, innovative solutions for delivery of self-management should be a priority. Unfortunately, despite the combination of numerous asthma apps being available and adolescents being early adopters of technology, with smartphones and tablets almost always at their fingertips, only seven out of 157 asthma apps identified in a systematic review in 2015 were targeted towards children or their parents, with none targeted specifically towards adolescents [40]. Since this systematic review in 2015, there have been only a small numbers of applications which have been designed for adolescents and young people identified in the literature [11,55,59,60,61]. Moreover, despite adolescents perceiving the use of smartphone and tablets as beneficial, particularly for symptom monitoring, and findings that it can be a feasible delivery mechanism for self-management skills and communication assistance with health-care professionals, adolescents with asthma are not commonly involved in the design process of many of these apps, with only one study clearly involving young people in the design process from the outset [5,58,59].

In a survey conducted in 2016 of over 1000 teenagers, 21% had downloaded a health-related app, however almost half (47%) of this group hardly ever or never used them [57]. This may be reflective of the level of difficulty in app use, or low interest and tailoring for the user. Involving adolescents in the creation is vital for a user-based perspective to ensure it is appealing and engaging. A recent systematic review in 2019 reviewed adolescents’ preferences for app features in health-related applications and identified an ability to customise, enhanced engagement through gamification, social media linking and observing their health trends were important to this population [62]. To our knowledge, only five studies over the last five years have reviewed adolescents’ preferences for content and features of health technology in asthma (Table 1) [4,5,26,63,64]. The use of tracking capabilities of symptoms and medications, medication reminders to promote adherence, and specific knowledge surrounding individual action plans and medication use have been identified as important components for teenagers with asthma [26].

Despite the lack of apps created specifically for adolescents, there are a small number of recent studies assessing the effects of apps for smartphone and tablet devices on adolescents with asthma, which have identified this as a feasible method to deliver education and self-management interventions [11,55,61,65,66]. Burbank et al. in 2015 found statistically significant improvement in asthma control in adolescents with uncontrolled asthma from baseline with the use of a personalised mobile asthma action plan on a smartphone app. This app provided feedback based on their personalised action plan in real time, based on the participants’ current symptoms or peak flow measurements. After using the personalised mobile asthma action plan for eight weeks, median Asthma Control Test (ACT) scores improved from 16 to 18 [11]. Perry et al. also reviewed the effects of a smartphone-based asthma action plan on a smartphone app, and found a statistically significant improvement in asthma control in those with uncontrolled asthma post-intervention, also using ACT scores [55]. In addition to a personalised asthma action plan on the app, medication and prescription refill reminders, as well as self-management tips and symptom/peak flow diaries were also available [55]. A proof-of-concept study by Mosnaim et al. in 2015, also showed promising results, with an electronic medication monitor and smartphone asthma app designed for a low health literacy population. The app in this study used gamification techniques and offered rewards such as monetary incentives for inhaled corticosteroid use by the participant. From baseline compared with completion of the eight-week treatment phase, the study found improvements in medication adherence (using an electronic medication monitoring device) as well as asthma control (using ACT scores) [61]. In 2019, a randomised controlled trial with an interactive app in which participants randomised to the intervention had access to for six months also showed improvement in adherence to inhaled corticosteroids in patients with a low baseline adherence [66]. This app had multiple features including disease control scoring, reminders for medication, informative videos, the ability to chat to peers in the study or a pharmacist, as well as fortnightly questions surrounding adherence [66]. Lastly, in 2021, Davis et al. undertook a short pilot study (six weeks) on an asthma application designed for young people (15–24 years old) and reported on significant changes in the ‘emotional function’ domain score of asthma quality of life (using the Mini Asthma Quality of Life Questionnaire) with no other statistically significant changes in other domains.

There has been only one systematic review to our knowledge, which reviews smartphone applications for asthma self-management specifically in adolescents by Alquran et al. in 2018. This combined interventional, observational and qualitative studies and identified smartphone apps as being able to have a positive effect on adolescents’ control and adherence to medications as well as self-efficacy; however, due to the small number and heterogeneity of included studies, no definitive conclusion on effect could be made [15].

## 7. Future Directions

mHealth may be a generation-appropriate, widely accessible and cost-effective solution for self-management interventions and education for adolescents, which is a known period for poorer asthma control. We have identified the following gaps in the current literature which we believe require addressing in order for successful implementation and use of smartphone and tablet apps used for self-management in adolescents with asthma.

### 7.1. Smartphone and Tablet Applications Designed Specifically for Adolescents Required

Several studies have demonstrated usefulness of smartphone and tablet applications for self-management in other chronic diseases, and in adults with asthma. Recent studies have also identified the feasibility of using smartphone and tablet devices as educational and self-management interventions in the adolescent asthma population. However, only five reports in the literature describe applications specifically designed for the adolescent asthma population. This infers a lack of evidence-based applications for adolescents are available. For successful implementation within the adolescent population, applications must be designed to be specific to the user [11,55,59,60,61].

### 7.2. Incorporation of Adolescents’ Preferences for Content and Features of Smartphone and Tablet Applications in Asthma Required and Co-Design Process Utilised

To ensure successful design, qualitative research must be undertaken to determine preferences, acceptability, and usability. Only one study to date has described implementing the co-design process, i.e., with contributions from the adolescent population, from the outset of application development, which we have identified as a major gap in development [59]. Intervention developers should be designing mHealth technology using co-design, and using existing evidence of adolescents’ preferences which we have highlighted in this review paper, to ensure successful engagement.

### 7.3. Larger Efficacy Studies Required

As mentioned, there has only been one systematic review to date which has analysed smartphone applications for asthma self-management in adolescents. Within the review, the included studies had a small number of participants with heterogeneity in both the study methods themselves, as well as in the intervention applied. Given the heterogeneity, no definitive conclusion could be made [15]. In view of this, larger efficacy studies are needed to identify the impact that this technology can have on asthma-related health outcomes for young people and to establish an evidence base that can launch a revolution in asthma self-management education.

mHealth creates an exciting opportunity for engaging adolescents during a busy time of their lives, using devices that the majority would have daily access to and use frequently. The incorporation of the co-design process to develop adolescent specific smartphone and tablet applications for self-management in asthma requires further attention, as we look to improve health outcomes in this vulnerable population.

## Figures and Tables

**Table 1:** Adolescents’ preferences for content and features of apps in asthma.

Author	Year	Study Design	Key Preferences Identified
Carpenter et al.	2016	Observational—usage of two asthma self-management apps over one week period by adolescents	Ability to increase self-observation and self-judgement via reminders, self-check quizzes, charting and tracking features such as symptoms and triggers Ability to share data and information from app with family, healthcare providers, school
Schneider et al.	2016	Observational—usage of two asthma apps over 7–10 days by adolescents	Ability to receive messages through the app, including reminders for medications, measuring peak flow, attend appointments Ability to receive alerts if deteriorating peak flow Motivational and supportive messages Visual aids (graphs/charts) to monitor trends of asthma status Ability to enter data such as peak flow Ability to share information and communicate with healthcare providers and others Customisation (e.g., ‘personally designed avatar’) and age-appropriate graphics Gamification
Roberts et al.	2018	Observational—usage of two asthma apps over one week by adolescents	Ease of use Simple layout Visual aids such as graphs and pictures Customisation—including medication lists, triggers, visuals and graphics of apps Gamification Ability to track asthma control Medication reminders Appointment reminders Information with videos
Ramsey et al.	2019	Qualitative	Ability to track symptoms Ability to track treatment and medications Reminders for medications, appointments Ability to deliver information on asthma and self-management (personalised medications and asthma plans) Be customisable to fit with daily life Ability to share data and information with health care providers and parents
Schneider et al.	2020	Qualitative, exploratory—asthma self management app trialled for 3 months by adolescents	Reminders for medications or checking peak flow Option to share information with health care provider Ability to review asthma patterns over time Age friendly visuals and graphics of app including backgrounds, fonts Ability to customise Availability of asthma training material e.g., videos on self-management strategies Ability to interact and communicate with others (healthcare providers, other teenagers, technological support) ‘Fun’ elements including gamification and incentives

## Data Availability

No new data were created or analyzed in this study. Data sharing is not applicable to this article.
